# *Pneumocystis* Pneumonia in Non-HIV Pregnant Women Receiving Chemotherapy for Malignant Lymphoma: Two Case Reports

**DOI:** 10.1155/2017/1073146

**Published:** 2017-08-28

**Authors:** Yuki Fukutani, Yoshitsugu Chigusa, Eiji Kondoh, Kaoru Kawasaki, Shingo Io, Noriomi Matsumura

**Affiliations:** Department of Gynecology and Obstetrics, Kyoto University, 54 Shogoin Kawahara-cho, Sakyo-ku, Kyoto 606-8507, Japan

## Abstract

*Pneumocystis* pneumonia (PCP) is a life-threatening opportunistic infection that sometimes occurs in immunocompromised patients with human immunodeficiency virus (HIV). Here, we report two extremely rare cases of PCP in non-HIV pregnant women who underwent chemotherapy for malignant lymphoma. Case  1 is a 34-year-old primigravida who was diagnosed with Hodgkin's lymphoma. She received ABVD chemotherapy and developed PCP at 37 weeks of gestation. After the onset of PCP, emergent cesarean section was performed due to a nonreassuring fetal status. Case  2 is a 31-year-old multigravida with diffuse large B-cell lymphoma who was administered R-CHOP chemotherapy. At 34 weeks of gestation, she complained of dyspnea and developed PCP. She delivered her baby vaginally immediately after the onset of symptoms. Both patients were treated with sulfamethoxazole-trimethoprim (ST) and recovered shortly thereafter. The babies' courses were also uneventful. PCP remains a serious cause of death, especially in non-HIV patients, and, therefore, appropriate prophylaxis and a prompt diagnosis are imperative.

## 1. Introduction

*Pneumocystis* pneumonia (PCP) is one of the most prevalent opportunistic infections in immunocompromised patients, especially in those infected with the human immunodeficiency virus (HIV). Lately, the incidence of PCP in non-HIV immunosuppressed patients has also been on the rise. These cases include hematological malignancies and patients who undergo chemotherapy for cancer or immunosuppressive drugs. In terms of PCP during pregnancy, most cases are HIV immunosuppressed patients, and non-HIV cases are extremely rare. Importantly, compared with HIV-positive patients, PCP in HIV-positive patients is associated with a higher mortality rate and poorer prognosis [1]. However, the standard prophylaxis and strategy for the treatment of PCP that develops in HIV-negative pregnant women have not yet been established.

This article describes two cases of PCP during pregnancy that occurred in patients receiving chemotherapy due to malignant lymphoma. We also reviewed the relevant literature to reveal the proper management of this life-threatening opportunistic infection in HIV-negative pregnant women.

## 2. Case Reports

### 2.1. Case  1

**Figure 1:**
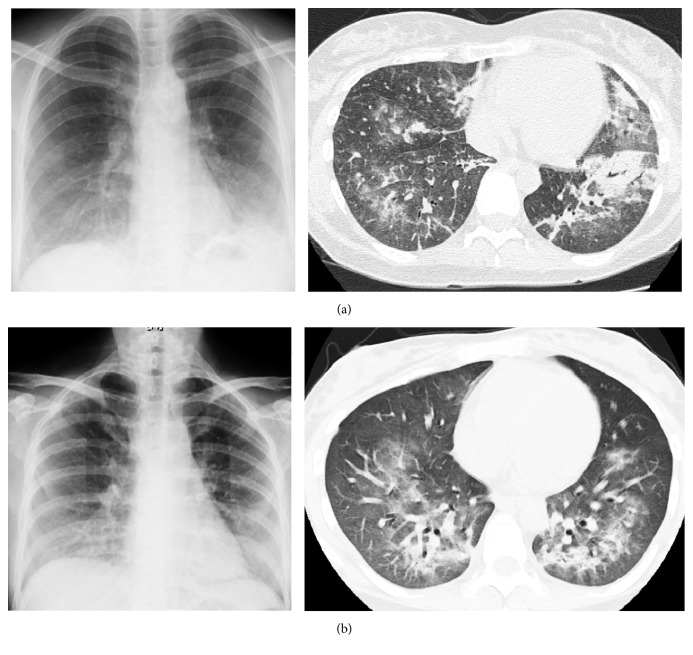
Chest X-ray (left) and computed tomography (CT) (right) images of case  1 (a) and case  2 (b). Chest X-ray showed a high density area in the lower lobes, which was detected as a diffuse ground-glass pattern on CT in both cases.

A 34-year-old woman, gravida 0, complained of a swollen lymph node on the left side of the neck and underwent a biopsy of the neck and mediastinal lymph nodes at 20 weeks of gestation. Pathological diagnosis was classical stage IIA (Ann Arbor staging system) Hodgkin's lymphoma, with involvement of the left supraclavicular and mediastinal nodes. ABVD (doxorubicin 25 mg/m^2^, bleomycin 10 mg/m^2^, vinblastine 6 mg/m^2^, and dacarbazine 375 mg/m^2^) chemotherapy was started at 23 weeks of gestation. After 4 cycles of ABVD, at 37 weeks and 0 days of gestation, she experienced a cough and slight dyspnea. Chest X-ray showed high density areas in both lower lobes, which were detected as diffuse ground-glass patterns on computed tomography (CT) (Figure 1(a)). We included interstitial pneumonia caused by bleomycin as well as PCP in the differential diagnosis, and oral prednisone was administered. We did not prescribe sulfamethoxazole-trimethoprim (ST) because the safety of ST in pregnancy was unclear at that time. At 37 weeks and 1 day of gestation, fetal movement was profoundly decreased, and repeated variable deceleration was observed on the fetal heart rate monitor. Consequently, emergent cesarean section due to nonreassuring fetal status and breech presentation was performed, and she delivered a 2596 g male baby. After surgery, she was treated with ST based on the clinical suspicion of PCP. Eventually, she was diagnosed with PCP by a PCR method that detects* Pneumocystis jirovecii*-specific DNA in the sputum. After a 3-week treatment with ST and prednisone, she recovered and thereafter received two additional cycles of ABVD. She has had no sign of disease relapse, and the subsequent clinical course of the baby has been uneventful.

### 2.2. Case  2

A 31-year-old woman, gravida 1, para 1, who had undergone conization due to stage IA1 uterine cervical cancer, noticed swelling of the right side of the pharynx shortly after she conceived; as a result, she visited an otolaryngologist. The tonsil biopsy at 13 weeks of gestation showed diffuse large B-cell lymphoma (DLBCL). The patient had multiple swollen lymph node groups on the same side of the diaphragm and was therefore classified as stage II according to the Cotswold modification of the Ann Arbor staging system. In all, 6 cycles of R-CHOP (rituximab 375 mg/m^2^, cyclophosphamide 750 mg/m^2^, doxorubicin 50 mg/m^2^, vincristine 1.4 mg/m^2^, and prednisone 100 mg/body) every 3 weeks were administered. At 32 weeks of gestation, she was hospitalized due to rapidly shortening cervical length (15 mm), and tocolysis with ritodrine was started. At 34 weeks and 0 days of gestation, she suddenly complained of dyspnea and a nonproductive cough, and chest CT showed a ground-glass pattern on both sides of the lungs (Figure 1(b)).* Pneumocystis* pneumonia was highly suspected in view of her history and CT findings, and she was presumptively treated with sulfamethoxazole-trimethoprim (ST), azithromycin, and oral prednisone. Then, tocolysis was stopped and she delivered a 1939 g female baby at 34 weeks and 1 day of gestation. Although* Pneumocystis jirovecii* was not observed in bronchoalveolar lavage (BAL) fluid by Grocott stain,* Pneumocystis jirovecii*-specific DNA was detected by PCR using BAL fluid. She recovered and was discharged home 14 days later, and her baby's course was uneventful. She has shown no signs of DLBCL recurrence.

## 3. Discussion

In the present report, we described two cases of PCP during pregnancy. Numerous cases of PCP in HIV-infected pregnant individuals have been reported. However, our cases are extremely rare in that both occurred in pregnant women without HIV. To the best of our knowledge, only one similar case of PCP has been reported thus far, and, in that case, PCP developed in a pregnant woman who was infected with human T lymphotropic virus type 1 (HTLV-1) [2].

With increasing maternal age, the incidence of cancer and the opportunity to administer chemotherapy during pregnancy also increase. Malignant lymphoma, which is the fourth most common malignancy diagnosed in pregnancy, is estimated to occur in 1 : 6,000 pregnancies [3]. Basically, chemotherapy in pregnant women with lymphoma should be similar to that in nonpregnant individuals, although it remains unclear whether the increased plasma volume and renal clearance of drugs during pregnancy might necessitate different doses of chemotherapy. The available existing data suggest that ABVD for Hodgkin's disease and CHOP for non-Hodgkin's disease during the second and third trimester can be administered safely without severe fetal outcomes [4]. In addition, compared with CHOP, rituximab with CHOP (R-CHOP) was revealed to be more effective and to lead to a better prognosis in patients (aged 18 to 60 years) with DLBCL [5]. Rituximab, which is a monoclonal antibody against CD20, is considered to be relatively safe for pregnant women although it may be associated with preterm birth [6, 7]. Based on these views, ABVD was given from 23 weeks of gestation in case  1, and R-CHOP was given from 16 weeks of gestation in case  2. In terms of R-CHOP, however, to our knowledge, only nine patients thus far have received that particular therapy [6–8], and more cases are necessary to analyze the safety of R-CHOP in pregnancy.

Generally, chemotherapy treatment for cancer can cause opportunistic infections, and patients with hematologic malignancies are particularly susceptible to a different set of infections [9]. However, the occurrence of PCP in patients who are administered R-CHOP or ABVD is infrequent. Hardak et al. reported that the incidence of PCP among patients treated with R-CHOP was 2.6% [10] in spite of the finding that R-CHOP includes a high dose of prednisone. Moreover, the frequency of PCP in patients who are given ABVD is currently unknown. The only literature that we found involved five patients with PCP and Hodgkin's lymphoma who were treated with ABVD [11]. Nevertheless, we have encountered two cases of PCP following ABVD or R-CHOP therapy. Presumably, PCP is more likely to occur in pregnant women who undergo cancer chemotherapy since pregnancy itself can give rise to immunosuppression [12].

Although PCP during pregnancy in HIV-negative patients is very rare, it is notable that the mortality rate of patients with PCP in the absence of HIV is higher (30 to 60%) than that in patients with HIV (10 to 20%) [13]. Moreover, PCP in HIV-negative patients has an abrupt onset that consists of respiratory insufficiency, and patients can deteriorate rapidly. Therefore, the appropriate prophylaxis is necessary to reduce the risk of PCP, even among HIV-negative pregnant women. Sulfamethoxazole-trimethoprim (ST), also known as cotrimoxazole, has already been demonstrated to be effective for the prevention of PCP in patients with HIV [14], while ST has been recommended for HIV-infected pregnant women with a low CD4 cell count. According to the systematic review and meta-analysis regarding the safety of cotrimoxazole conducted by Ford et al., the overall prevalence of congenital abnormalities in those exposed to cotrimoxazole was not significantly higher than the reported rates in the general population [15]. Thus, patients with hematological cancers, such as lymphoma and leukemia, are at risk for PCP because they are treated with cytotoxic chemotherapy, which sometimes causes immunosuppression. In addition, chronic corticosteroid administration is one of the most common risk factors for PCP in patients without HIV infection. For nonpregnant women, therefore, it is recommended that prophylaxis with ST be considered for those with hematological malignancies who are treated with chemotherapy, and for those who are treated with more than 20 mg/day of prednisone for longer than 1 month [16]. This recommendation may be pertinent to pregnant women as well.

In addition to prophylaxis, prompt diagnosis and appropriate treatment are essential for a good prognosis of PCP. However, PCP is sometimes difficult to diagnose, as the patients present nonspecific symptoms such as fever, dyspnea, and nonproductive cough. Although the final diagnosis of PCP should be made based on the microscopic detection of* Pneumocystis* from specimens of sputum or bronchoalveolar fluid, the PCR method that is used to detect* Pneumocystis jirovecii*-specific nucleic acids has also become available, which has a higher diagnostic sensitivity. Moreover, chest CT is more sensitive and superior as a diagnostic tool compared with chest radiography. Indeed, in our two cases, expeditious CT revealed typical features of PCP, such as a ground-glass attenuation, which encouraged us to initiate treatment with sulfamethoxazole-trimethoprim. The fetal radiation doses from chest CT are very low, since this type of scan does not involve direct irradiation, but rather it results in scattered radiation. The maximum estimated conceptus radiation dose from chest CT is less than 1 mGy [17], and the average dose is 0.22 mGy [18]. Therefore, physicians should not hesitate to perform chest CT examination, even during pregnancy, when PCP is suspected.

In summary, we described two rare cases of PCP after chemotherapy was administered for lymphoma during pregnancy. PCP remains a serious cause of death in immunocompromised patients, and, therefore, appropriate prophylaxis and prompt diagnosis are imperative.
